# Hospital Readmissions Among Infants With Neonatal Opioid Withdrawal Syndrome

**DOI:** 10.1001/jamanetworkopen.2024.35074

**Published:** 2024-09-24

**Authors:** Julie R. Gaither, Matthew J. Drago, Matthew R. Grossman, Yi Li, Veronika Shabanova, Xiao Xu, John M. Leventhal

**Affiliations:** 1 Department of Pediatrics, Yale School of Medicine, New Haven, Connecticut; 2 Department of Chronic Disease Epidemiology, Yale School of Public Health, New Haven, Connecticut; 3 Division of Newborn Medicine, Icahn School of Medicine at Mount Sinai, New York, New York; 4 Department of Biostatistics, Yale School of Public Health, New Haven, Connecticut; 5 Department of Obstetrics and Gynecology, Vagelos College of Physicians and Surgeons, Columbia University Irving Medical Center, New York, New York

## Abstract

**Importance:**

Although cases of neonatal opioid withdrawal syndrome (NOWS) increased 5-fold in recent years, no study has examined national hospital readmission rates for these infants.

**Objective:**

To examine hospital readmissions for infants with and without NOWS.

**Design, Setting, and Participants:**

This retrospective cohort study analyzed serial cross-sectional samples of US hospital discharge records from the Nationwide Readmissions Database for calendar years 2016 to 2020. Infants with NOWS were identified using *International Classification of Diseases, Tenth Revision, Clinical Modification* codes. The data analysis was performed between January 5, 2023, and May 6, 2024.

**Exposure:**

Neonatal opioid withdrawal syndrome.

**Main Outcome and Measures:**

Survey-weighted logistic regression was used to examine 90-day all-cause and cause-specific hospital readmissions. Multivariable models adjusted for sex, low birth weight, gestational age, multiple gestation, type of insurance, and year of birth.

**Results:**

Of the 13 855 246 newborns identified in this weighted analysis, 89 018 (0.6%) were diagnosed with NOWS, of whom 53.8% were male and 81.1% born full-term (>36 weeks gestation). The 90-day all-cause readmission rate was 4.2% for infants with NOWS compared with 3.0% for those without NOWS (*P* < .001). After risk adjustment, the odds of all-cause readmission were higher among infants with NOWS (adjusted odds ratio [AOR], 1.18; 95% CI, 1.08-1.29). Infants with NOWS had significantly higher odds of readmissions for seizures (AOR, 1.58; 95% CI, 1.01-2.46), failure to thrive (AOR, 1.99; 95% CI, 1.36-2.93), traumatic brain injury (AOR, 2.95; 95% CI, 1.76-4.93), and skull fractures (AOR 3.72; 95% CI, 2.33-5.93). Infants with NOWS had higher odds of receiving a diagnosis of confirmed maltreatment (AOR, 4.26; 95% CI, 2.19-8.27), including for neglect (AOR, 14.18; 95% CI, 5.55-36.22) and physical abuse (AOR, 2.42; 95% CI, 0.93-6.29); however, the latter finding was not statistically significant.

**Conclusions and Relevance:**

In this nationally representative cohort study, infants with NOWS were at increased risk of readmission for any cause as well as for trauma and confirmed maltreatment. These findings may in part reflect the dual stressors that mothers with opioid use disorder face in caring for a newborn with NOWS in the context of a substance use disorder and underscore the need for family-based, in-home services that focus concurrently on substance use treatment and parenting support.

## Key Points

**Question:**

Does the risk of hospital readmission differ between infants with and without neonatal opioid withdrawal syndrome (NOWS)?

**Findings:**

In this cohort study of 13 855 246 newborns, infants with NOWS had a 90-day readmission rate of 4.2% compared with 3.0% for infants without NOWS. After risk adjustment, infants with NOWS were more likely to be readmitted for serious injuries and confirmed neglect but not physical abuse.

**Meaning:**

These findings suggest that infants with NOWS are at an increased risk of 90-day readmission for trauma and maltreatment.

## Teaser

This cohort study uses current US hospital discharge data to examine readmission rates of infants with and without neonatal opioid withdrawal syndrome.

## Introduction

Over the past 20 years, the increased use of prescribed and illicit opioids among women of child-bearing age (15-44 years) in the US has resulted in a 5-fold increase in neonatal opioid withdrawal syndrome (NOWS).^1,2^ During the birth hospitalization, infants with NOWS, compared with full-term newborns without complicating conditions, are more likely to experience central nervous system irritability, respiratory distress, intolerance to feedings, and a number of other adverse events during withdrawal,^1,2,3,4,5,6^ resulting in a median length of stay of 12 days vs less than 2 days for all other hospital births.^5,7^

Far less is known about hospitalizations for infants with NOWS beyond the immediate postpartum period, particularly on a national level. The few studies that have been published relied on data from selected states and hospitals and have suggested that infants with NOWS remain medically complex and vulnerable following the initial hospital discharge and have substantially higher rates of all-cause readmissions than other newborns.^8,9,10^ The generalizability of these studies, however, is limited by several factors, including data from more than a decade ago, differences in comparison groups, and known variations across states in the care of infants with NOWS. This variation has only intensified over the past decade with the development of new models of care, such as the Eat, Sleep, Console (ESC) approach.^11,12,13^

Regarding the causes of readmission, the literature indicates that infants with NOWS continue to experience many of the same conditions they experienced during the birth hospitalization.^8^ Moreover, in a population-based study in New South Wales, Australia, NOWS was found to be associated with readmissions for maltreatment (ie, neglect or abuse) during childhood.^14^ Puls et al^15^ found that newborns exposed to alcohol or drugs in utero had higher rates of 6-month readmissions than newborns who had not been exposed, but their study was limited to readmissions for physical abuse only. In contrast, Austin et al^16^ found that while infants exposed prenatally to substances were more likely to receive a diagnosis of neglect, they were less likely to receive a diagnosis of abuse.

Given the need to understand at a national level the risk of hospital readmission for this vulnerable population, our objective was to build on prior research by examining recent (2016-2020) all-cause and cause-specific readmissions using nationally representative longitudinal data on infants with NOWS. The findings may provide a better understanding of postdischarge outcomes for infants with NOWS, particularly for rare events, such as maltreatment.

## Methods

### Study Overview

In this retrospective cohort study, we used a nationally representative sample of US discharge records for calendar years 2016 through 2020 to compare 90-day hospital readmissions for infants with NOWS with those of all other newborns. The data were deidentified and considered exempt from approval and informed consent by the institutional review board of the Yale School of Medicine. This study followed the Strengthening the Reporting of Observational Studies in Epidemiology (STROBE) reporting guideline for cohort studies.

### Study Design and Data Source

We used the Nationwide Readmissions Database (NRD), a nationally representative sample of discharge records developed by the Agency for Healthcare Research and Quality for the Healthcare Cost and Utilization Project.^17^ The NRD is released annually and includes hospital discharge data drawn from Healthcare Cost and Utilization Project State Inpatient Databases for 1 calendar year for inpatient admissions and readmissions, regardless of payer. For this study, we used 2016 (first full year with *International Classification of Diseases, Tenth Revision, Clinical Modification* [*ICD-10-CM*] coding) through 2020 (most recently available data). In 2016, 27 geographically diverse states contributed data to the NRD, whereas 31 states contributed data in 2020. Discharge weights are designed based on hospital location, ownership, teaching status, and number of beds to generate nationally representative estimates. The NRD uses unique patient linkage numbers to track patients across hospitalizations that occur within 1 calendar year (patients cannot be tracked across multiple years or across state lines). Discharge records were excluded if patient linkage numbers were missing or unverified (ie, same patient linkage number but different date of birth and/or sex). For patients younger than 1 year, only states with verified patient linkage numbers on at least 90% of discharge records were included in the NRD datasets. As some states do not report on patients younger than 1 year to the NRD, the NRD compensates by applying a higher weight to available discharges for these patients. Thus, in 2016 and 2020, respectively, 7.0% and 6.4% of State Inpatient Database discharges were excluded for children younger than 1 year.

### Identification of Infant Cohorts and Main Outcome Measures

As shown in the Figure, we identified 18 442 499 weighted (4 003 752 unweighted) newborns in the 2016-2020 NRD by first limiting the sample to those younger than 1 year who also had an *ICD-10-CM* code for liveborn infants. The eTable in Supplement 1 shows the specific *ICD-10-CM* codes used for identifying infants and conditions. For these infants, we then selected the first hospitalization record for the relevant calendar year. Infants who died during the birth hospitalization were excluded. To allow for equal 90-day follow-up, our primary analysis was limited to newborns discharged from the birth hospitalization between January and September of each calendar year. In sensitivity analyses of all-cause readmissions, we included newborns discharged between January and December. We also compared infants discharged before or in September and those discharged after September of a given year.

**Figure.**
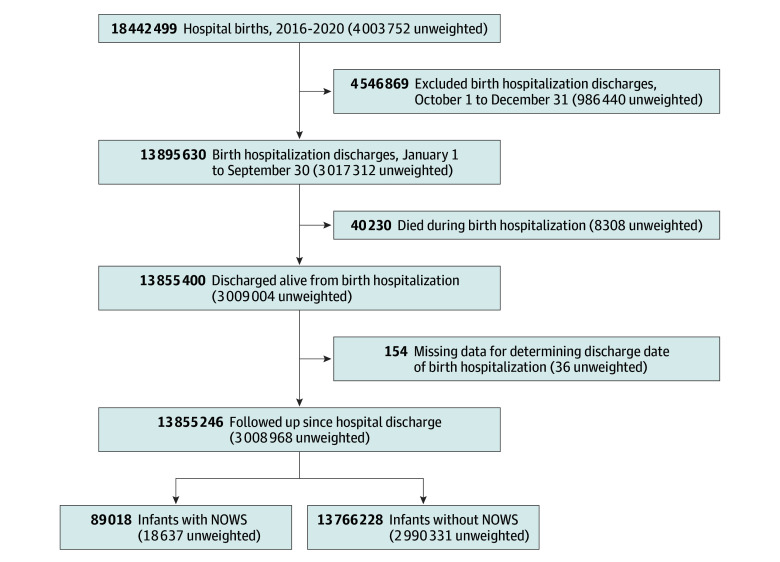
Flow Diagram of Sample Selection NOWS indicates neonatal opioid withdrawal syndrome.

To assess the incremental impact of NOWS, we used *ICD-10-CM* code P96.1 (infants with NOWS [neonatal withdrawal symptoms from maternal use of drugs of addiction]) to create a binary indicator of NOWS as the key exposure variable.^18^ (Note that to remain consistent with the term currently used by the American Academy of Pediatrics and federal agencies, including the US Food and Drug Administration, we refer to the condition traditionally known as neonatal abstinence syndrome as NOWS.^19,20^)

The *ICD-10-CM* codes were also used to identify multiple gestation births and infants born with low birth weight (<2500 g), early preterm (ie, gestational age <34 weeks), or late preterm (gestational age between 34 weeks, 0 days and 36 weeks, 6 days).^21^ Full-term newborns were defined by the absence of a prematurity code.

The index date for determining 90-day readmission was the discharge date of the birth hospitalization. For cause of readmission, we used the principal diagnosis code and all secondary diagnosis codes^22^ and chose a priori to focus on medical problems common to infants with NOWS noted in prior research.^1,3,23^ For determining whether infants died during a readmission, we used the discharge disposition information in the NRD data.

### Statistical Analysis

Data were analyzed between January 5, 2023, and May 6, 2024. To account for the complex survey design of the NRD, all analyses incorporated sampling weights and strata and cluster variables.

Descriptive statistics were used to characterize the sample based on weighted national estimates. Race and ethnicity variables are not available in the Healthcare Cost and Utilization Project dataset and were therefore not analyzed. Differences in demographic and clinical characteristics and readmission rates for infants with NOWS vs infants without NOWS were assessed using χ^2^ tests for categorical variables and, as appropriate, *t* or Wilcoxon rank sum tests for continuous variables.

Survey-weighted logistic regression was used to estimate unadjusted and adjusted odds ratios (AORs) for readmissions overall and by cause. For the adjusted analyses, based on clinical expertise and a review of the literature,^8,9,10,15^ we chose a priori to control for sex, low birth weight, gestational age, singleton vs multiple gestation, type of insurance, and year of the birth hospitalization. Unless otherwise noted, we report the weighted estimates and adjusted results.

To test the robustness of our findings overall (due to the restriction of births from January to September each year), we conducted a sensitivity analysis using Cox proportional hazards regression to examine the hazard ratio (HR) for the number of days to readmission when all newborns were included, regardless of discharge month. In a separate sensitivity analysis, we examined the HR of readmission for infants discharged after September of each year.

All analyses were completed using SAS, version 9.4 software (SAS Institute, Inc). Because each individual hypothesis was independent, we did not adjust the α level for multiple comparisons.^24^ *P* < .05 indicated statistical significance.

## Results

### Birth Hospitalization

Of the 13 855 246 newborns identified in this weighted analysis, 89 018 (0.6%) were diagnosed with NOWS (vs 13 766 228 [99.4%] without NOWS) (Figure). Most infants were born full-term (aged >36 weeks) (with NOWS, 81.1%; without NOWS, 91.1%) and were male (with NOWS, 53.8% vs 46.2% female; without NOWS, 51.3% vs 48.7% female). Table 1 compares key sociodemographic characteristics of the birth hospitalization for infants with and without a NOWS diagnosis.

**Table 1.** Sociodemographic Characteristics for Infants by NOWS Diagnosis for the Birth Hospitalization, 2016-2020 (N = 13 855 246)

Characteristic	No. of infants (%)^a^		*P* value
	With NOWS (n = 89 018)	Without NOWS (n = 13 766 228)	
Sex			
Female	41 098 (46.2)	6 710 828 (48.7)	<.001
Male	47 920 (53.8)	7 055 400 (51.3)	<.001
Birth weight <2500 g	7841 (8.8)	487 391 (3.5)	<.001
Gestational age range			
Early preterm (<33 completed wk)	3291 (3.7)	325 152 (2.4)	<.001
Late preterm (34-36 completed wk)	13 531 (15.2)	896 850 (6.5)	
Full-term (>36 completed wk)	72 196 (81.1)	12 544 227 (91.1)	
Multiple gestation (vs singleton)	2189 (1.8)	540 942 (3.0)	<.001
Birth at metropolitan teaching hospital	66 365 (74.6)	9 793 005 (71.1)	<.001
Birth hospitalization length of stay, median (IQR), d	11.3 (4.6-20.3)	1.6 (1.1-2.2)	<.001
Type of insurance			
Private	9382 (10.6)	6 606 419 (48.0)	<.001
Medicaid	74 382 (83.7)	6 101 453 (44.4)	
Self-pay	3737 (4.2)	560 038 (4.1)	
Other	1409 (1.6)	483 669 (3.5)	

Abbreviation: NOWS, neonatal opioid withdrawal syndrome.

^a^ All numbers reflect weighted national estimates. Percentages may not sum to 100 due to rounding.

### All-Cause 90-Day Readmission

The 90-day all-cause readmission rate was 4.2% for infants with NOWS compared with 3.0% for infants without NOWS (*P* < .001). Thus, the unadjusted OR of readmission was higher for infants with NOWS compared with infants without NOWS (1.42; 95% CI, 1.30-1.55). After risk adjustment, infants with NOWS had higher odds of being readmitted within 90 days for any cause (AOR, 1.18; 95% CI, 1.08-1.29).

As shown in Table 2, of the 412 320 infants who were readmitted at least once, 3693 with NOWS had a median length of stay for the first readmission of 2.3 days (IQR, 1.2-6.4 days) compared with 1.6 days (IQR, 0.8-3.1 days) for the 408 627 infants without NOWS (*P* < .001). The proportion of newborns readmitted more than once within 90 days was similar for infants with NOWS to those without NOWS (7.3% vs 6.4%, respectively; *P* = .30).

**Table 2.** Health Care Use Characteristics for Readmitted Infants by NOWS Diagnosis, 2016-2020 (n = 412 320)

Characteristic	With NOWS (n = 3693)	Without NOWS (n = 408 627)	*P* value
Time since initial discharge to first readmission, median (IQR), d	23.7 (8.7-43.6)	14.4 (2.3-37.7)	<.001
Length of stay at first readmission, median (IQR), d	2.3 (1.2-6.4)	1.6 (0.8-3.1)	<.001
Readmissions within 90 d, No. (%)^a^			
1	3422 (92.7)	382 671 (93.7)	.30
≥2	271 (7.3)	25 957 (6.4)	
Disposition of patient for first readmission, No. (%)^a^			
Routine	3430 (93.6)	396 799 (97.2)	<.001
Home with health care services	163 (4.4)	7696 (1.9)	
Transfer to short-term hospital or other medical facility	70 (1.9)	3921 (1.0)	

Abbreviation: NOWS, neonatal opioid withdrawal syndrome.

^a^ All numbers reflect weighted national estimates. Percentages may not sum to 100 because of rounding errors.

### Causes of Readmission

Table 3 shows that the absolute number of readmissions for each condition examined was low, and the proportion of infants admitted was less than 0.5% for both infants with and without NOWS. However, even after risk adjustment, infants with NOWS compared with those without NOWS had lower odds for readmission for sepsis (AOR, 0.24; 95% CI, 0.10-0.61) but higher odds for readmission for seizures (AOR, 1.58; 95% CI, 1.01-2.46) and failure to thrive (AOR, 1.99; 95% CI, 1.36-2.93). There was no difference in readmission odds for feeding difficulties (AOR, 1.08; 95% CI, 0.84-1.40) or respiratory disorders (AOR, 1.08; 95% CI, 0.81-1.43).

**Table 3.** Clinical Characteristics of Readmissions for Infants by NOWS Diagnosis, 2016-2020 (N = 13 855 246)^a^

Characteristic	No. of infants (%)		OR (95% CI)	
	With NOWS (n = 89 018)	Without NOWS (n = 13 766 228)	Unadjusted	Adjusted^b^
Clinical problems				
Feeding difficulties	415 (0.47)	51 640 (0.38)	1.25 (0.97-1.60)	1.08 (0.84-1.40)
Respiratory disorders	361 (0.41)	39 803 (0.29)	1.41 (1.06-1.86)	1.08 (0.81-1.43)
Sepsis	16 (0.02)	8490 (0.06)	0.30 (0.12-0.74)	0.24 (0.10-0.61)
Seizures	108 (0.12)	8054 (0.06)	2.07 (1.33-3.23)	1.58 (1.01-2.46)
Failure to thrive	166 (0.19)	9783 (0.07)	2.63 (1.80-3.84)	1.99 (1.36-2.93)
Any injury	408 (0.46)	19 978 (0.15)	3.17 (2.48-4.04)	2.26 (1.77-2.89)
Any head injury	183 (0.21)	5957 (0.04)	4.76 (3.30-6.86)	3.43 (2.37-4.94)
Traumatic brain injury	96 (0.11)	3812 (0.03)	3.92 (2.35-6.53)	2.95 (1.76-4.93)
Skull fracture	102 (0.11)	3204 (0.02)	4.95 (3.12-7.85)	3.72 (2.33-5.93)
Anoxic brain injury or asphyxia	31 (0.03)	931 (0.01)	5.18 (2.39-11.23)	3.28 (1.49-7.25)
Falls	109 (0.12)	2664 (0.02)	6.31 (3.96-10.05)	5.30 (3.33-8.41)
ALTE in infancy	117 (0.13)	13 952 (0.10)	1.30 (0.74-2.30)	0.88 (0.50-1.56)
Child maltreatment	140 (0.16)	3399 (0.02)	6.40 (4.04-10.12)	3.87 (2.45-6.11)
Suspected	85 (0.10)	2188 (0.02)	6.03 (3.37-10.81)	3.57 (1.99-6.39)
Confirmed	55 (0.06)	1259 (0.01)	6.77 (3.47-13.21)	4.26 (2.19-8.27)
Neglect or abandonment	30 (0.03)	165 (<0.01)	20.28 (10.36-77.34)	14.18 (5.55-36.22)
Physical abuse	25 (0.03)	1042 (0.01)	3.68 (1.43-9.50)	2.42 (0.93-6.29)

Abbreviations: ALTE, apparent life-threatening event; NOWS, neonatal opioid withdrawal syndrome; OR, odds ratio.

^a^ All numbers reflect weighted national estimates.

^b^ Models adjusted for sex, low birth weight, gestational age, multiple gestation, type of insurance, and year of birth.

Compared with infants without NOWS, infants with NOWS had higher odds of being readmitted for any head injury (AOR, 3.43; 95% CI, 2.37-4.94), traumatic brain injury (AOR, 2.95; 95% CI, 1.76-4.93), and skull fracture (AOR, 3.72; 95% CI, 2.33-5.93). In addition, infants with NOWS had higher odds of readmission for anoxic brain injury or asphyxia (AOR, 3.28; 95% CI, 1.49-7.25) and falls (eg, off furniture or from someone’s arms) (AOR, 5.30; 95% CI, 3.33-8.41).

Infants with NOWS had higher odds than infants without NOWS of receiving a diagnosis of suspected or confirmed maltreatment upon readmission (AOR, 3.87; 95% CI, 2.45-6.11). The AOR of readmission for confirmed maltreatment was 4.26 (95% CI, 2.19-8.27) for infants with NOWS. Whereas infants with NOWS had higher odds of readmissions for confirmed neglect (AOR, 14.18; 95% CI, 5.55-36.22), there was no statistical difference in readmissions for confirmed physical abuse (AOR, 2.42; 95% CI, 0.93-6.29).

### Fatalities

Among readmitted infants (n = 412 320), only 29 (0.8%) with NOWs and 1720 (0.4%) without NOWS died during a readmission. The mortality odds did not differ significantly between the 2 groups, and the nonsignificance persisted after risk adjustment (AOR, 1.48; 95% CI, 0.66-3.33). The exact cause of death could not be clearly determined solely from *ICD-10-CM* codes because cardiac arrest or respiratory failure were listed as the principal or secondary diagnosis code for all deaths.

### Sensitivity Analyses

Sensitivity analysis including all births (regardless of discharge month) (n = 18 422 499) showed similar results to the main analyses. The HR for all-cause readmission was 1.37 (95% CI, 1.27-1.48) for infants with NOWS. For infants discharged after September of a given year, the HR for readmission was 1.24 (95% CI, 1.06-1.45) for infants with NOWS.

## Discussion

To our knowledge, this cohort study is the first to use the latest nationally representative data to comprehensively examine all causes of hospital readmissions for infants born with NOWS. There are 3 key findings from this analysis of hospital discharge records representing nearly 14 million newborns: (1) infants with NOWS had a 90-day all-cause readmission rate of 4.2% compared with 3.0% for infants without NOWS; (2) after risk adjustment, readmissions for failure to thrive, seizures, and serious injuries, including head injuries, were substantially higher in infants with NOWS; and (3) infants with NOWS were more likely to be readmitted with a diagnosis of confirmed maltreatment from neglect but not physical abuse.

Our findings based on national data are consistent with previous regional and hospital-based studies.^8,9,10,25^ Specifically, Patrick et al^8^ examined 30-day readmissions in the state of New York between 2006 and 2009 and found that infants with NOWS had a readmission risk of 3.0% compared with 3.7% for late-preterm births and 1.9% for uncomplicated full-term births. In an analysis of pediatric hospital data, Milliren et al^10^ found that 9.9% of infants with NOWS were readmitted within 1 year. In these previous studies, however, the adjusted risk of readmission was 1.5 to 2.5 times higher for infants with NOWS,^8,9,10^ which is notably higher than our adjusted results. These studies examined different readmission time frames and comparison groups and included data from approximately 1 decade earlier than those we used. Our up-to-date national data showed that of the 5 medical conditions we examined, only 2 led to early readmissions (ie, within 90 days) for infants with NOWS: seizures and failure to thrive.

We found the largest differences in readmissions for serious physical injuries and maltreatment. These findings are consistent with the results of a recent study that relied on 2013 and 2014 national data and showed that infants exposed to alcohol or drugs prenatally and who had neonatal abstinence syndrome were 3 times more likely than unexposed newborns to be readmitted within 6 months for a physical injury due to child maltreatment.^15^ The authors also found that infants exposed to alcohol or drugs and who had symptoms of withdrawal during the newborn hospitalization were at an increased risk of future physical abuse. In contrast, in a study using Medicaid data from 3 states, Austin et al^16^ found that while infants exposed to substances were more likely than unexposed infants to receive a diagnosis of neglect, they were less likely to receive a diagnosis of physical abuse. We, in turn, found that infants with NOWS were 14 times more likely than infants without NOWS to be readmitted within 90 days with a diagnosis of confirmed neglect, suggesting a substantial risk of neglect in this population. Similar to Austin et al,^16^ we did not find a statistically significant difference in readmissions for physical abuse.

In part, the injury and maltreatment findings may be explained by maternal and family stress associated with caring for a vulnerable newborn in the context of a substance use disorder.^23,26,27,28,29^ These findings may also reflect the complexity on the part of health care professionals, hospitals, and child protective services of delivering safe discharge planning to families given the intensive health care resources needed to care for infants with NOWS during the birth hospitalization and the substantial strain that has been placed on institutions in recent years due to maternal opioid use.^5^ A 2013 study showed that child protective services workers in Massachusetts were dedicating more than 10 000 hours per month specifically to infants with opioid exposure, resulting in an estimated $4.3 million in labor costs for the state,^30^ costs that have not been met with increased funding. In general, the current US health care and child welfare systems remain underfunded and inadequately equipped to provide the in-home, comprehensive services that are likely needed to reduce NOWS-associated readmissions and comorbidities.^31,32,33,34^ Given the complex challenges that families affected by maternal opioid use face once they leave the hospital with their newborns, further investment in the expansion of home-based models of care that focus concurrently on substance use treatment, parenting support, and early intervention services might benefit both mother and child.^32,33,34,35^

Standardization of postdischarge care is also needed on a national level.^36^ The Child Abuse Prevention and Treatment Act was amended in 2016 to ensure plans of safe care for infants with opioid exposure or withdrawal, but how those plans have been interpreted and implemented has varied widely across states.^37^ A national system that would allow for monitoring state data and comparing it with national averages may identify the most effective discharge processes.

Further research is needed to understand how the treatment of mothers with substance use disorders during the birth hospitalization may leave them ill-prepared to care for their newborns after discharge. Although extensive improvements^13^ have been made in managing withdrawal for infants with NOWS during the birth hospitalization using the ESC approach to care,^11,38^ which promotes parental involvement, breastfeeding, rooming in, and palliative comfort measures, there remains substantial variation in the uptake and implementation of this model nationwide.^39^ Many infants with NOWS continue to be treated using the Finnegan approach^40,41^ (or a modified version), which prioritizes pharmacologic treatment of withdrawal symptoms and the delivery of care in neonatal intensive care units. Thus, mothers are both separated from their newborns and discouraged from breastfeeding, each of which may present barriers to developing a relationship with their newborn. Moreover, given the complexity of care and the intensity of resources needed to manage withdrawal in infants with NOWS, many mothers may not receive the same newborn teaching (eg, safe sleep) from health care professionals that other mothers are given. The stigma that many mothers with substance use disorders experience after giving birth may further limit health-seeking behaviors and engagement with the health care system.^42^ Research is also needed to understand what role bias may play in the diagnosis of maltreatment, as anecdotal evidence suggests that mothers with substance use disorders may not be granted the benefit of the doubt when it comes to events that would not normally be considered maltreatment.

### Limitations

This study has several limitations. First, as previously mentioned, some states do not report on patients aged younger than 1 year to the NRD. However, to compensate for this limited reporting, the NRD applies a higher weight to available discharge data on these patients, still allowing for assessment of national estimates.^43^ Second, our measurement of clinical conditions is based on *ICD-10-CM* codes, which are subject to potential miscoding, overcoding, and errors of omission. A study validating the use of administrative data for measuring NOWS, however, found that the *ICD-10-CM* code P96.1, which we used in our study, has a high positive predictive value of 91%.^44^ Reliance on *ICD-10-CM* codes to identify cases of child neglect^45^ and physical abuse is particularly challenging. Thus, we may have undercounted the number of maltreatment cases in this sample. Third, as with any study that relies on administrative data, the extent to which we were able to examine the clinical or social profile of the child and family was limited. Fourth, because there is no state identifier in the NRD, we were unable to control for the variation across states in the care of infants with NOWS^39^ (eg, the association of ESC with length of stay during the birth hospitalization and subsequent readmissions).^13,38,46,47^ Fifth, each year of the NRD data was constructed separately. Thus, we could not track patients across years, limiting our ability to measure longer-term readmissions. Finally, in choosing a control group to serve as a comparator for infants with NOWS, there was no clear choice that stood out as ideal. We felt that the most straightforward method was to compare infants with NOWS with those without NOWS.

## Conclusions

In this cohort study, we found that infants with NOWS had a higher risk of all-cause 90-day hospital readmission than other newborns and were at a substantially increased risk of readmission for trauma and maltreatment associated with neglect but not physical abuse. These findings reflect the complex psychosocial factors that may not only lead to opioid use disorder but also complicate parenting of infants with NOWS in their initial months at home, highlighting a need for improved funding and support for postdischarge medical and welfare services. Family-based, in-home services that focus concurrently on substance use treatment and parenting support may be particularly beneficial.

## Notes

Supplement 1. **eTable.** *International Classification of Diseases, Tenth Revision, Clinical Modification* (*ICD-10-CM*) Codes to Identify Infants Characteristics and Main Outcome Measures

Supplement 2.
Data Sharing Statement
