# Supplementary material for: Hospital Readmissions Among Infants With Neonatal Opioid Withdrawal Syndrome
Source: JAMA Netw Open. 2024 Sep 24;7(9):e2435074. doi: 10.1001/jamanetworkopen.2024.35074 (PMC11423163; doi:10.1001/jamanetworkopen.2024.35074)
Supplement: Supplement 1. — eTable. International Classification of Diseases, Tenth Revision, Clinical Modification (ICD-10-CM) Codes to Identify Infants Characteristics and Main Outcome Measures [file jamanetwopen-e2435074-s001.pdf]

Supplemental Online Content

Gaither JR, Drago MJ, Grossman MR, et al. Hospital readmissions among infants with neonatal opioid withdrawal syndrome. *JAMA Netw Open*. 2024;7(9):e2435074. doi:10.1001/jamanetworkopen.2024.35074

eTable. *International Classification of Diseases, Tenth Revision, Clinical Modification* (*ICD-10-CM*) Codes to Identify Infants Characteristics and Main Outcome Measures

This supplemental material has been provided by the authors to give readers additional information about their work.

© 2024 Gaither JR et al. *JAMA Network Open*.

eTable. ***International Classification of Diseases, Tenth Revision, Clinical Modification*** **(*ICD-10-CM*) Codes to Identify Infants Characteristics and Main Outcome Measures**

**Description**	***ICD-10-CM* Code**
Liveborn infant	Z38.x
Multiple gestation (e.g., twins, multiple births)	Z38.3-.8
Infants with NOWS (neonatal withdrawal symptoms from maternal use of drugs of addiction)	P96.1
Low birth weight (< 2,500 grams)	P07.16-.18
Gestational age	
< 23 completed weeks	P07.21
23 completed weeks	P07.22
24 completed weeks	P07.23
25 completed weeks	P07.24
26 completed weeks	P07.25
27 completed weeks	P07.26
28 completed weeks	P07.31
29 completed weeks	P07.32
30 completed weeks	P07.33
31 completed weeks	P07.34
32 completed weeks	P07.35
33 completed weeks	P07.36
34 completed weeks	P07.37
35 completed weeks	P07.38
36 completed weeks	P07.39
Feeding problems of newborn	P92.x

© 2024 Gaither JR et al. *JAMA Network Open*.

Respiratory disorders	P22.x-P28.x
Sepsis	P36.x
Seizures	P90.x and R56.x
Failure to thrive	P92.6
Any injury	S00-T34, T36-T50, T67-T71, T79
Any head injury	S00-S09
Traumatic brain injury	S06
Skull fracture	S02
Anoxic brain injury	G93.1
Asphyxiation	T71
Falls	W0.0-W0.4, W0.6-W0.9, W10, W18, W19
Apparent life-threatening event	R68.13
Child maltreatment	
Suspected	T76.x
Confirmed	T74.x
Neglect or abandonment	T74.02
Physical abuse	T74.12, T74.4

© 2024 Gaither JR et al. *JAMA Network Open*.
