# Supplementary material for: Hospital Readmissions Among Infants With Neonatal Opioid Withdrawal Syndrome
Source: JAMA Netw Open. 2024 Sep 24;7(9):e2435074. doi: 10.1001/jamanetworkopen.2024.35074 (PMC11423163; doi:10.1001/jamanetworkopen.2024.35074)
Supplement: Supplement 2. — Data Sharing Statement [file jamanetwopen-e2435074-s002.pdf]

Data Sharing Statement

Gaither. Hospital Readmissions Among Infants With Neonatal Opioid Withdrawal Syndrome. *JAMA Netw Open*. Published September 24, 2024. doi:10.1001/jamanetworkopen.2024.35074

Data
Data available: Yes
Data types: Deidentified participant data
How to access data: This study involved deidentified data obtained from the Healthcare Cost and Utilization Project: https://hcup-us.ahrq.gov. Data are publicly available.
When available: With publication

Supporting Documents
Document types: Other (please specify)
Additional Information: Data are publicly available.
How to access documents: https://hcup-us.ahrq.gov.
When available: With publication

Additional Information
Who can access the data: Data are currently available at the HCUP website.
Types of analyses: Per HCUP criteria.
Mechanisms of data availability: Data are publicly available.
